# Supplementary material for: A Humanized Yeast Phenomic Model of Deoxycytidine Kinase to Predict Genetic Buffering of Nucleoside Analog Cytotoxicity
Source: Genes (Basel). 2019 Sep 30;10(10):770. doi: 10.3390/genes10100770 (PMC6826991; doi:10.3390/genes10100770)
Supplement: Supplementary file 1 [file genes-10-00770-s001.zip › Additional_Files/Additional_File7_GOTermHeatmaps.zip!/Additional_File7_GOTermHeatmaps/phosphorylation_of_RNA_polymerase_II_C-terminal_domain.pdf]

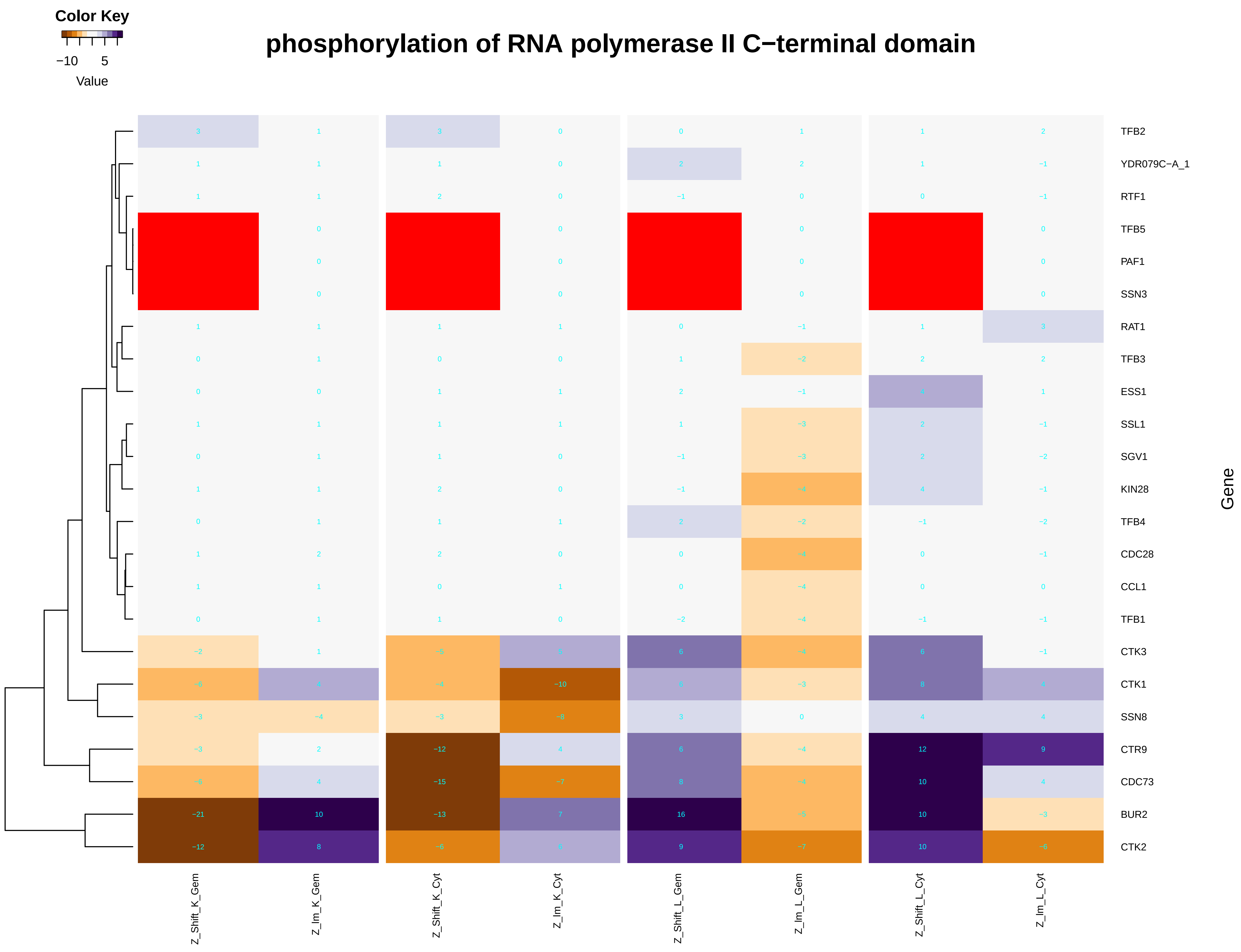

Color Key
−10
5
Value
phosphorylation of RNA polymerase II C−terminal domain
TFB2
YDR079C−A_1
RTF1
TFB5
PAF1
SSN3
RAT1
TFB3
ESS1
SSL1
SGV1
KIN28
TFB4
CDC28
CCL1
TFB1
CTK3
CTK1
SSN8
CTR9
CDC73
BUR2
CTK2
Gene
Z_Shift_K_Gem
Z_Im_K_Gem
Z_Shift_K_Cyt
Z_Im_K_Cyt
Z_Shift_L_Gem
Z_Im_L_Gem
Z_Shift_L_Cyt
Z_Im_L_Cyt

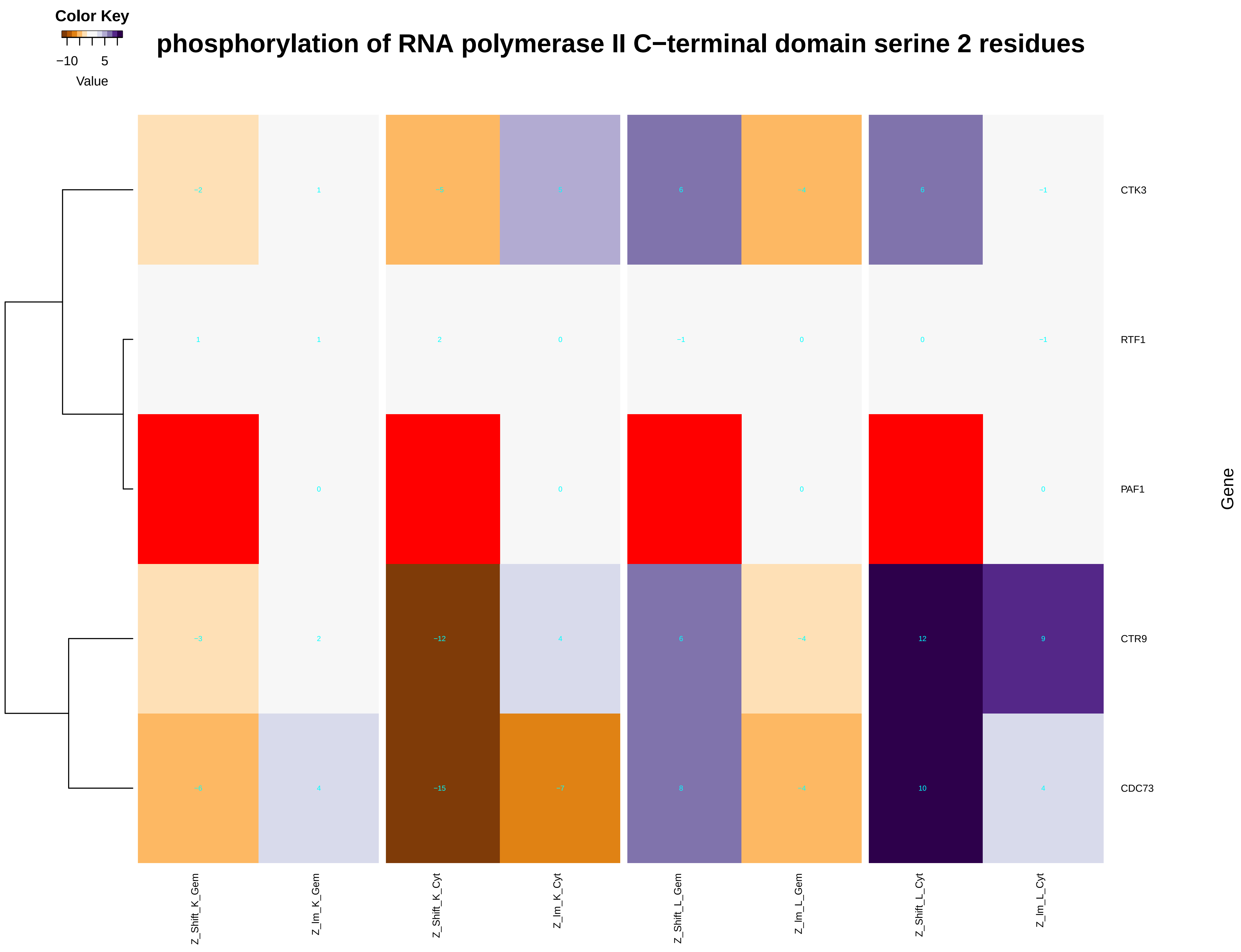
Color Key
−10
5
Value
phosphorylation of RNA polymerase II C−terminal domain serine 2 residues
CTK3
RTF1
PAF1
CTR9
CDC73
Gene
Z_Shift_K_Gem
Z_Im_K_Gem
Z_Shift_K_Cyt
Z_Im_K_Cyt
Z_Shift_L_Gem
Z_Im_L_Gem
Z_Shift_L_Cyt
Z_Im_L_Cyt
